# Consumption of whole and refined grains and the risk of gastric cancer: a pooled analysis within the Stomach cancer Pooling (StoP) Project

**DOI:** 10.1007/s00394-026-04003-w

**Published:** 2026-06-13

**Authors:** Federica Turati, Francesca Bravi, Claudio Pelucchi, Rossella Bonzi, Kenneth C. Johnson, Jinfu Hu, Monica Ferraroni, Zuo Feng Zhang, Guopei Yu, Nuno Lunet, Samantha Morais, David Zaridze, Dmitry Maximovitch, Jesus Vioque, Sandra Gonzalez-Palacios, Maria Paula Curado, Emmanuel Dias-Neto, Gemma Castaño-Vinyals, Nerea Fernández de Larrea-Baz, Lizbeth López-Carrillo, Raul Ulises Hernández-Ramirez, Shoichiro Tsugane, Gerson Shigueaki Hamada, Mary H. Ward, Lina Mu, Charles S. Rabkin, Akihisa Hidaka, Areti Lagiou, Pagona Lagiou, Malaquias López-Cervantes, Paolo Boffetta, M. Constanza Camargo, Stefania Boccia, Eva Negri, Carlo La Vecchia

**Affiliations:** 1 Department of Clinical Sciences and Community Health, Dipartimento di Eccellenza 2023-2027, Università degli Studi di Milano, via Celoria 22, Milan, 20133 Italy; 2 School of Epidemiology and Public Health, Department of Medicine, University of Ottawa, Ottawa, Canada; 3 Harbin Medical University, Harbin, China; 4 Ospedale Maggiore Policlinico, Fondazione IRCCS Ca’ Granda, Milan, Italy; 5 Department of Epidemiology, UCLA Fielding School of Public Health and Jonsson Comprehensive Cancer Center, Los Angeles, CA USA; 6 Medical Informatics Center, Peking University, Peking, China; 7 EPIUnit ITR, Instituto de Saúde Pública da Universidade do Porto, Porto, Portugal; 8 Departamento de Ciências da Saúde Pública e Forenses e Educação Médica, Faculdade de Medicina da Universidade do Porto, Porto, Portugal; 9 Department of Clinical Epidemiology, N.N.Blokhin National Medical Research Center for Oncology, Moscow, Russia; 10 Instituto de Investigación Sanitaria y Biomédica de Alicante, Universidad Miguel Hernandez (ISABIAL-UMH), Alicante, Spain; 11 Consortium for Biomedical Research in Epidemiology and Public Health (CIBERESP), Madrid, Spain; 12 Centro Internacional de Pesquisa, A. C. Camargo Cancer Center, São Paulo, Brazil; 13 Rutgers Cancer Institute, Rutgers University, Newark, NJ USA; 14 ISGlobal, Barcelona, Spain; 15 Universitat Pompeu Fabra (UPF), Barcelona, Spain; 16 National Center for Epidemiology, Instituto de Salud Carlos III, Madrid, Spain; 17 National Institute of Public Health, Morelos, Mexico; 18 Department of Biostatistics, Yale School of Public Health, Yale School of Medicine, New Haven, CT USA; 19 Division of Cohort Research, National Cancer Center Institute for Cancer Control, Tokyo, Japan; 20 International University of Health and Welfare Graduate School of Public Health, Tokyo, Japan; 21 Nikkei Disease Prevention Center, São Paulo, Brazil; 22 Division of Cancer Epidemiology and Genetics, National Cancer Institute, National Institutes of Health, Rockville, MD USA; 23 Department of Epidemiology and Environmental Health, School of Public Health and Health Professions, University at Buffalo, Buffalo, NY USA; 24 Division of Epidemiology, National Cancer Center Institute for Cancer Control, Tokyo, Japan; 25 Department of Diabetes and Endocrinology, JCHO Tokyo Yamate Medical Centre, Tokyo, Japan; 26 Department of Public and Community Health, School of Public Health, University of West Attica, Athens, Greece; 27 Department of Hygiene, Epidemiology and Medical Statistics, School of Medicine, National and Kapodistrian University of Athens, Athens, Greece; 28 Department of Epidemiology, Harvard T.H. Chan School of Public Health, Boston, MA USA; 29 Facultad de Medicina, UNAM, Coyoacán, Mexico; 30 Department of Medical and Surgical Sciences, University of Bologna, Bologna, Italy; 31 Stony Brook Cancer Center, Stony Brook University, Stony Brook, NY USA; 32 University Department of Life Sciences and Public Health, Section of Hygiene, Università Cattolica del Sacro Cuore, Roma, Italy; 33 Department of Woman and Child Health and Public Health, Fondazione Policlinico Universitario A. Gemelli IRCCS, Rome, Italy

**Keywords:** Case-control studies, Consortium, Diet, Gastric cancer, Pooled analysis

## Abstract

**Purpose:**

The relationship between whole and refined grain intake and gastric cancer risk has been investigated, but findings remain inconclusive. We aimed to evaluate and quantify the association of whole and refined grain consumption with gastric cancer risk through an individual participant pooled analysis of studies participating in the Stomach cancer Pooling (StoP) Project.

**Methods:**

Twenty case-control studies (including 7,943 cases and 19,729 controls) contributed to the analysis of refined grains, and 13 of these (5,658 cases and 15,802 controls) contributed to the analysis of whole grains. Study-specific odds ratios (ORs) were estimated using multivariable logistic regression models and pooled through a two-stage approach based on fixed-effects models.

**Results:**

For whole grains, compared with no consumption, the OR was 0.92 (95% confidence interval, CI: 0.84–1.01) for any consumption, and 0.86 (95% CI: 0.77–0.96) for a consumption equal or above the study-specific median. There was an increasing risk of gastric cancer with increasing consumption of refined grains, with an OR for the highest versus the lowest tertile of 1.39 (95% CI: 1.28–1.50) when considering staple grain foods only and 1.52 (95% CI: 1.39–1.65) when also considering grain-based sweets and desserts.

**Conclusion:**

Our findings indicate that whole grain consumption is inversely associated, and refined grain consumption directly associated with gastric cancer risk. These findings support current dietary recommendations favoring whole grains over refined grains.

**Supplementary Information:**

The online version contains supplementary material available at 10.1007/s00394-026-04003-w.

## Introduction

Grains are staple foods worldwide, representing a major source of carbohydrates and plant protein and accounting for approximately 50% of the daily caloric intake [1]. They are largely consumed in their refined form, in which the bran and germ are removed. In contrast, whole grains retain the entire kernel, including the bran, germ, and endosperm, with abundant nutrients and bioactive compounds, including fiber, antioxidants, vitamins and polyphenols.

Whole grain consumption has been associated with a reduced risk of total mortality and several non-communicable diseases, including cardiovascular disease, type 2 diabetes, and certain types of cancer, particularly colorectal cancer [2–5]. Regarding gastric cancer, available epidemiological evidence suggests a potential protective effect of whole grain consumption and a possible increased risk associated with refined grain consumption [6–8]; however, findings remain inconclusive [9].

The Stomach cancer Pooling (StoP) Project [10] is an international consortium of epidemiologic studies on gastric cancer risk factors. Access to individual-level data within the consortium enables individual participant data pooled analyses, which, compared to standard meta-analyses, allow for harmonized exposure definitions and consistent covariate adjustment. The StoP Project, therefore, provides a unique opportunity to investigate this topic in greater depth.

In the present work, we evaluated and quantified the association of whole and refined grain consumption with gastric cancer risk within the StoP Project.

## Methods

### Population

The present analysis is based on the third data release of the StoP Project consortium (http://www.stop-project.org/), which includes 34 case–control and nested case–control studies of gastric cancer conducted worldwide [10]. The StoP Project is a global consortium of epidemiological studies of gastric cancer aiming at assessing the role of several lifestyles and genetic determinants in the etiology of gastric cancer by pooled analyses of individual participant data (http://www.stop-project.org/). Details on the StoP Project methods have been given elsewhere [11]. The original data from each study were obtained based on a data transfer agreement between the principal investigators and the coordinating centre. All data were harmonized according to a prespecified format at a centralised pooling centre at the University of Milan. The StoP Project received ethical approval from the University of Milan Review Board (reference 19/15 on 01/04/2015).

For the present work, we selected 20 case-control studies in which the food frequency questionnaire (FFQ) collected individual grain food items and whose investigators agreed to participate in the analysis, for a total of 7,943 cases and 19,729 controls. There were 8 studies from Europe (3 from Italy [12–14], 1 from Greece [15], 2 from Spain [16, 17], 1 from Portugal [18], 1 from Russia [19]), 4 from Asia (3 from China [20–22] and 1 from Japan [23]), 2 from North America (1 from the USA [24] and 1 from Canada [25]), and 6 from Central-South America (3 from Brazil [26–28] and 3 from Mexico [29–31]. Of these, 13 [12–19, 21, 24, 25, 31, 32] collected data on whole grains (5,658 cases and 15,802 controls) (Supplementary Table 1).

### Grain consumption

In all studies, grain consumption was assessed using FFQs. The consumption of refined grains and whole grains was obtained by summing up the consumption of the food items included in the corresponding food group (e.g., white rice, pasta, and white bread for refined grains; whole grain bread, brown rice, oatmeal, and whole wheat pasta for whole grains). For complex mixed dishes containing grains (e.g., pizza, lasagna), the grain component was estimated based on standard recipe proportions. Consumption was expressed in portions per week; when the consumption was provided in grams, the weight reported was converted into portions considering standard portion size from nutritional databases. To account for the distinct nutritional profiles of staple grain foods (e.g., bread, cereals, pasta, white rice) and indulgent grain foods (e.g., cakes, cookies, doughnuts, brownies, muffins, pastries) - the latter being higher in added sugars, saturated fats and energy density - we derived two separate variables for refined grain consumption: one including only staple grain foods (named “refined grains”, thereafter) and one including both staple and indulgent grain foods (“refined grains including sweets”, thereafter). The latter variable was derived in 17 out of 20 studies (7,358 cases and 18,784 controls) which collected consumption of grain-based sweets/desserts.

### Data analysis

Consumption of refined grains and of refined grains including sweets was categorized into study-specific tertiles. Whole grain consumption was categorized as no consumption, and above or below the study-specific median consumption (calculated among consumers), since the distribution of consumption did not allow meaningful computation of tertiles in several studies. In an additional analysis, high whole grain consumption was defined as ≥ 7 portions/week.

To quantify the association between grain consumption and gastric cancer, a two-stage modeling approach was used. First, study-specific odds ratios (OR) with corresponding 95% confidence intervals (CI) were estimated using multivariable logistic regression models, including terms for sex, age (generally in 5-year age groups, or study-specific categories when required by the data distribution), socioeconomic status (low, intermediate, or high, as defined in each original study based on education, income or occupation), tobacco smoking (never, former and current smokers of ≤ 10 cigarettes/day; 11 to 20 cigarettes/day; >20 cigarettes/day), alcohol drinking (never, low: ≤12 g of ethanol/day, intermediate: >12 to 47 g of ethanol/day, high: >47 g of ethanol/day), fruit and vegetable intake (low, intermediate, or high as defined, when possible, by study-specific tertiles), salt intake (low, intermediate, high), total energy intake (study-specific quintiles), and family history of gastric cancer (yes, no). Covariates that were not collected in a given study were not included in the corresponding study-specific models. Specifically, data were available in 11 studies for total energy intake (4,365 cases and 9,281 controls) (Supplementary Table 1), in 17 studies for salt intake, and in 14 studies for family history of gastric cancer. For covariates that were collected within a study but had missing values at the individual level, a separate dummy category was included in the model to retain those subjects in the analysis; when the number of missing values was too small to define a separate category, missing data were imputed using the study-specific modal category. At the second stage, summary ORs were derived by pooling the study-specific ORs using fixed-effects models. Heterogeneity across studies was quantified using the I^2^ statistic.

We tested for the significance of linear trends across categories of grain consumption by estimating study-specific trends (i.e., considering the variable as ordinal in the logistic model), and using the Wald test p values deriving from the pooled estimate.

We conducted selected sensitivity analyses: we recalculated the pooled ORs according to random-effects models based on the DerSimonian and Laird method; we used a one-stage approach and derived pooled estimates using generalized linear mixed effect models with logistic link function and random intercept for each study; we further adjusted for *Helicobater pylori* (*H. pylori*) infection in the 10 studies with available data (6 of those with whole grains data, Supplementary Table 1); and we re-calculated the pooled ORs by removing one study at a time from the two-stage procedure to assess the impact of individual studies on the summary result. Stratified analyses were conducted to evaluate whether the associations differed across subgroups defined by age, sex, geographic area, and type of controls (hospital-based, population-based). Difference between subgroups was assessed through the Q test for heterogeneity.

Analyses were performed using SAS version 9.4 (SAS Institute Inc., Cary, NC), and RStudio version 2024.4.2 (RStudio, Inc., Boston, MA, USA). P-values < 0.05 were considered statistically significant.

## Results

Supplementary Figure S1 presents the participant flow chart. Table 1 shows the main characteristics of the 7,943 cases of gastric cancer and 19,729 controls from the 20 case-control studies which contributed to the analysis on refined grains. Compared to controls, cases were somewhat older (≥ 65 years: 46.2% versus 39.8%), more frequently males (63.4% versus 55.2%), of low socioeconomic status (56.4% versus 41.9%), high and moderate current smokers (8.2% and 11.3% versus 6.5% and 9.6%, respectively), and heavy alcohol drinkers (16.6% versus 11.1%).

**Table 1:** Distribution of gastric cancer cases and controls contributing to the analysis of refined grain consumption within the Stomach cancer Pooling (StoP) Project consortium according to age, sex, and other selected covariates

	Cases		Controls	
	n*	%	n*	%
Age				
< 40	348	4.4	1626	8.3
40–44	330	4.2	1274	6.5
45–49	559	7.0	1706	8.7
50–54	753	9.5	1990	10.1
55–59	1026	12.9	2380	12.1
60–64	1255	15.8	2890	14.7
65–69	1480	18.6	3301	16.7
70–74	1389	17.5	2800	14.2
≥ 75	803	10.1	1751	8.9
Sex				
Male	5039	63.4	10,888	55.2
Female	2904	36.6	8841	44.1
Socioeconomic status				
Low	4414	56.4	8174	41.9
Intermediate	2402	30.7	6723	34.5
High	1005	12.9	4614	23.7
Smoking habits				
Never	3328	43.6	8947	46.4
Former	2258	29.6	5544	28.7
Current smokers, number cigarettes/day				
≤ 10	561	7.4	1697	8.8
11–20	861	11.3	1860	9.6
> 20	624	8.2	1245	6.5
Alcohol drinking (g/day)^				
Never	2116	30.5	5839	32.7
≤ 12	1528	22.0	5670	31.7
> 12–47	2149	31.0	4381	24.5
> 47	1149	16.6	1985	11.1
Fruit and vegetable intake				
Low	2447	31.6	5637	29.5
Intermediate	2558	33.1	6549	34.2
High	2728	35.3	6942	36.3
Salt intake^#^				
Low	2437	41.2	6679	39.6
Intermediate	1964	33.2	5678	33.6
High	1438	24.3	4419	26.1
Intermediate or high	71	1.2	113	0.7
Family history of gastric cancer^§^				
No	3390	80.8	9146	92.0
Yes	805	19.2	800	8.0

^*^ The sum may not add up to the total because of some missing values. ^Data not available for study China 3. ^#^ Data not available for the following studies: Italy 4, Greece, China 3. ^§^ Data not available for the following studies: China 1, Canada, China 3, Mexico 1, Mexico 2, Mexico 3

**Fig. 1:**
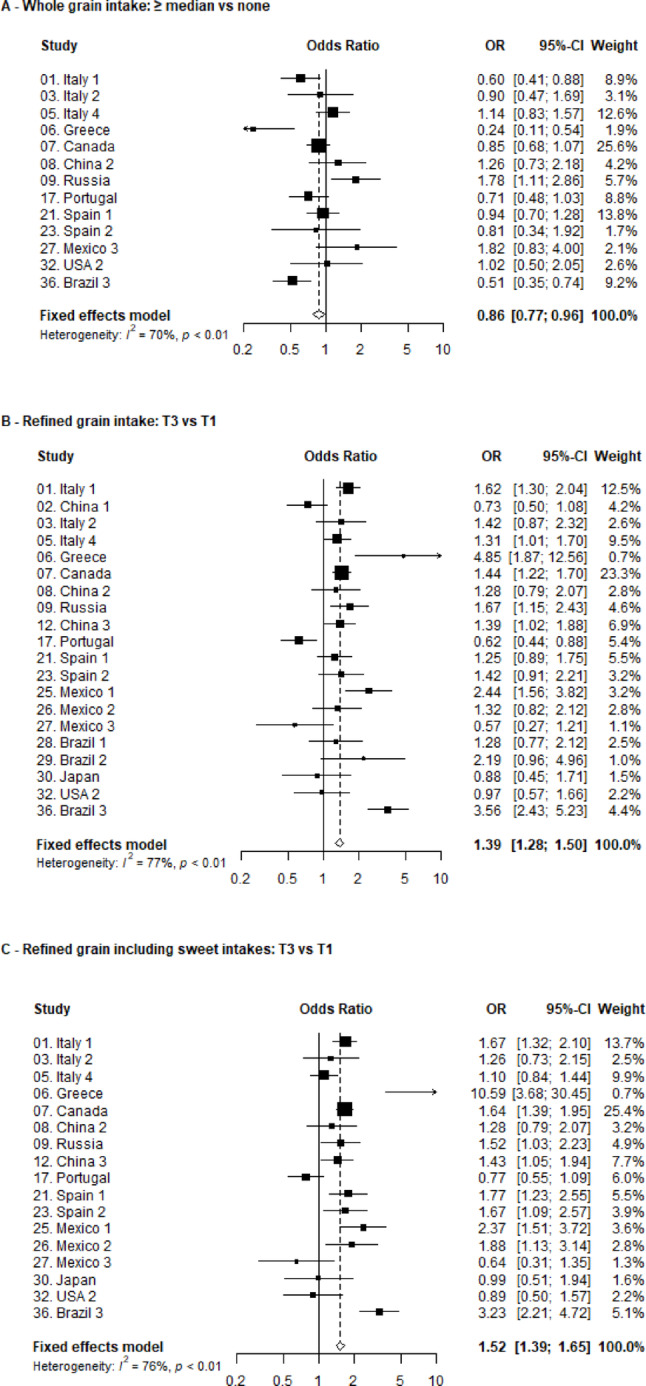
Forest plots showing study-specific and pooled odds ratios (OR)^ and the corresponding 95% confidence intervals (CI) of gastric cancer for the highest versus the lowest category of consumption of whole grains (i.e., ≥ study-specific median consumption versus no consumption,** A**), refined grains (third versus first study-specific tertile,** B**) and refined grains including sweets (third versus first study-specific tertile,** C**). ^Derived from multivariable logistic regression models including, when available and applicable, terms for sex, age, socioeconomic status, smoking, alcohol drinking, fruit and vegetable consumption, salt intake, total energy intake, and family history of gastric cancer.

Table 2 provides the pooled ORs and the corresponding 95% CIs of gastric cancer according to the consumption of whole and refined grains. The corresponding forest plots with study-specific and pooled ORs for the highest versus the lowest categories of consumption are provided in Fig. 1. Regarding whole grains, compared to no consumption, the ORs were 0.92 (95% CI: 0.84–1.01, I² = 66.7%) for any consumption, 0.99 (95% CI: 0.87–1.13, I² = 26.0%) for a consumption below the median, and 0.86 (95% CI: 0.77–0.96, I² = 69.6%) for a consumption equal or above the median. The OR was 0.88 (95% CI: 0.79-1.00, I² = 64.6%) for ≥ 7 portions per week. For refined grains, increasing risks were observed across tertiles of consumption. The OR for the highest versus the lowest tertile was 1.39 (95% CI: 1.28–1.50; I² = 76.6%) when considering only staple grain foods, and 1.52 (95% CI: 1.39–1.65; I² = 75.9%) when also considering the consumption of grain-based sweets and desserts. The former OR (1.39) became 1.42 (95% CI: 1.31–1.55) when restricting the analysis to the 17 studies contributing to the analysis on the variable refined grains including sweets. The associations persisted, but were reduced, when including the studies in which adjustment for total energy intake was possible (OR for the highest tertile: 1.21, 95% CI: 1.07–1.36, for refined grains, and 1.26, 95% CI: 1.11–1.42, for refined grains including sweets).

**Table 2:** Distribution of gastric cancer cases and controls according to consumption of whole and refined grains, pooled odds ratios^ (OR) and corresponding 95% confidence intervals (CI) of gastric cancer

	Cases		Controls		OR^^^ (95% CI)	I^2^ (%)
	n*	%	n*	%		
Whole grains [13 studies]						
No	3048	54.6	6865	43.7	Reference	
Yes	2538	45.4	8842	56.3	0.92 (0.84–1.01)	66.7
< median intake^¶^	1257	22.5	4368	27.8	0.99 (0.87–1.13)	26.0
≥median intake	1281	22.9	4474	28.5	0.86 (0.77–0.96)	69.6
*p trend*					*0.013*	
Refined grains [20 studies]						
Tertile^§^1	2209	28.1	6823	34.8	Reference	
Tertile 2	2652	33.8	6564	33.5	1.21 (1.12–1.30)	24.3
Tertile 3	2989	38.1	6235	31.8	1.39 (1.28–1.50)	76.6
*p trend*					*< 0.001*	
Refined grains including sweets^#^ [17 studies]						
Tertile^§^ 1	2017	27.8	6555	35.1	Reference	
Tertile 2	2430	33.4	6266	33.6	1.24 (1.15–1.35)	35.4
Tertile 3	2819	38.8	5857	31.4	1.52 (1.39–1.65)	75.9
*p trend*					*< 0.001*	

* The sum does not add up to the total because of some missing values. ^Derived from fixed-effects models meta-analyses which pooled study-specific ORs derived from multivariable logistic regression models including, when available and applicable, terms for sex, age, socioeconomic status, smoking, alcohol drinking, fruit and vegetable consumption, salt intake, total energy intake, and family history of gastric cancer. ^¶^Study-specific, calculated among consumers. ^#^ Studies China 1, Brazil 1 and Brazil 2 did not collect data on grain-based sweets and desserts, and did not contribute to the analysis. ^§^ Study-specific

In sensitivity analyses, the ORs derived from random-effects models were 0.87 (95% CI: 0.69–1.09) for whole grain consumption equal or above the median, 1.37 (95% CI: 1.14–1.64) for the third tertile of consumption of refined grains, and 1.51 (95% CI:1.25–1.82) for the third tertile of consumption of refined grains including sweets. The corresponding ORs from a one-stage approach were 0.89 (95% CI: 0.81–0.98), 1.34 (95% CI: 1.24–1.44), and 1.46 (95% CI: 1.35–1.56). Further adjustment for *H. pylori* infection in studies with available data did not materially affect the results, yielding corresponding ORs of 0.86 (95% CI: 0.77–0.96), 1.39 (95% CI: 1.28–1.51), and 1.53 (95% CI: 1.40–1.67), respectively.

The omission of any individual study did not alter the direction and magnitude of the observed effects. In particular, the pooled estimate ranged between 0.83 and 0.91 for whole grain consumption equal or above the median versus none, between 1.33 and 1.45 for the third versus first tertile of consumption of refined grains, and between 1.46 and 1.59 for the third versus first tertile of consumption of refined grains including sweets (Supplementary Figure S2).

Results from the stratified analyses are provided in Table 3. The association with refined grains was stronger in hospital-based than in population-based case-control studies, and in studies from the Americas.

**Table 3:** Pooled odds ratios (OR), with 95% confidence intervals (CI), of gastric cancer for the highest versus the lowest category of consumption of whole grains, refined grains, and refined grains including sweets in strata of selected factors

	Whole grains ≥median vs. none		Refined grains T3 vs. T1		Refined grains including sweets T3 vs. T1	
	OR (95% CI)	I^2^ (%)	OR (95% CI)	I^2^ (%)	OR (95% CI)	I^2^ (%)
Age						
≤ 60	0.77 (0.64–0.92)	62.1	1.45 (1.28–1.65)	66.8	1.65 (1.44–1.90)	56.0
> 60	0.94 (0.81–1.09)	34.1	1.32 (1.18–1.46)	58.9	1.42 (1.27–1.59)	65.9
*p for heterogeneity*	*0.095*		*0.267*		*0.099*	
Sex						
Male	0.87 (0.75–1.01)	64.6	1.32 (1.19–1.47)	53.9	1.51 (1.34–1.69)	48.8
Female	0.82 (0.69–0.99)	52.4	1.45 (1.27–1.66)	78.8	1.46 (1.27–1.68)	77.3
*p for heterogeneity*	*0.620*		*0.280*		*0.717*	
Geographic area						
Europe	0.89 (0.76–1.03)	73.2	1.34 (1.19–1.51)	77.2	1.40 (1.23–1.58)	78.9
Asia*	-		1.13 (0.91–1.39)	70.6	1.38 (1.07–1.79)	0.0
Americas	0.80 (0.67–0.95)	71.7	1.54 (1.36–1.74)	78.4	1.71 (1.50–1.95)	78.2
*p for heterogeneity*	*0.367*		*0.035*		*0.071*	
Type of controls						
Population-based	0.92 (0.81–1.06)	7.2	1.30 (1.18–1.44)	71.6	1.42 (1.28–1.58)	71.9
Hospital-based	0.75 (0.61–0.91)	80.6	1.53 (1.35–1.74)	79.7	1.73 (1.50–2.01)	78.9
*p for heterogeneity*	*0.097*		*0.048*		*0.032*	

^Derived from fixed-effects models meta-analyses which pooled study-specific ORs derived from multivariable logistic regression models including, when available and applicable, terms for sex, age, socioeconomic status, smoking, alcohol drinking, fruit and vegetable consumption, salt intake, total energy intake, and family history of gastric cancer. * The pooled estimate for Asian studies for whole grain intake was not calculated since only one study was available (study China 2)

## Discussion

The present analysis from the international StoP consortium, based on nearly 8,000 gastric cancer cases and 20,000 controls with data on grain consumption from 11 distinct countries worldwide - including approximately 5,600 cases and 15,800 controls with data on whole grains - found increasing gastric gastric risk with increasing tertiles of refined grain intake, with a stronger excess observed when grain-based sweets and desserts were considered in addition to staple grain foods. Whole grains were inversely related, with a 14% decreased risk for the highest consumption category; however, the latter estimate lost statistical significance under the random-effect model. The differential role of whole and refined grains may be explained by their complementary consumption, as individuals with high whole grain consumption are likely to consume lower amounts of refined grains, and vice versa.

In stratified analyses, the association with refined grains was stronger in studies using hospital-based compared to population-based controls, though the differences were of borderline significance. Refusal rates are typically higher in population-based than hospital-based case-control studies [33]. In population-based case-control studies with low response rates, the results of unfavorably related factors may be biased toward the null if participation among controls was associated with healthier lifestyle behaviors. Indeed, lower education, lower socio-economic status, smoking, low physical activity, and obesity are all associated with non-participation [34, 35].

For refined grains, geographical differences were observed, with a stronger association in American than European studies. While these differences may partly reflect variations in refined grain consumption patterns across regions, the heterogeneity of results observed even among studies conducted within the same countries/geographic areas – e.g., among the 4 Latin America studies and among the 2 studies from North America (as outlined in the forest plot with study-specific ORs) - weights against this explanation.

Our results are in line with those from previous meta-analyses, which reported an inverse association between whole grain consumption and gastric cancer risk [7, 8], and a direct one with refined grain consumption [7]. Specifically, based on 11 studies published up to March 2020 and including over 8,200 cases overall, Zhang et al. [8] estimated a pooled relative risk (RR) of gastric cancer of 0.64 (95% CI: 0.53–0.79) for the highest versus the lowest category of whole grain consumption, with a stronger association in case-control than cohort studies. The 2020 meta-analysis by Wang et al. [7], based on a different set of 5 studies, provided a pooled RR of 0.61 (95% CI: 0.43–0.85) for whole grain consumption of at least 3 times per week versus the study-specific lowest category. Based on 16 studies, the same meta-analysis estimated pooled RRs of 1.28 (95% CI: 1.18–1.39) and 1.63 (95% CI: 1.49–1.79) respectively for intermediate (i.e., 1–2 times per week) and high (i.e., at least 3 times per week) refined grain consumption.

Grains may influence gastric cancer risk through various mechanisms. Unlike their refined counterparts, whole grains retain the bran and germ fractions during processing, thereby preserving nutrients. Whole grains are rich in antioxidants, including vitamins (e.g., vitamin E, B1, B2, some carotenoids), trace minerals (e.g., selenium, copper, zinc), phenolic acids (e.g., ferulic acids, phytic acid, lignans), and phytoestrogens, which collectively reduce oxidative damage and stress, potentially decreasing cancer risk. Inverse associations were reported between the intake of phenolic acids [36], selenium [37], vitamin E [38], and lignans [39] and gastric cancer risk. Whole grains are also a rich source of fiber. Higher dietary fiber intake has been associated with reduced gastric cancer risk in several studies [40, 41], with some evidence that the protective effect may be driven by fiber from cereals [42–44]. Under acid conditions - similar to those that exist in the stomach - fiber can scavenge nitrite [45], a precursor for N-nitroso compounds, which has been linked with an increased risk of cancer [46], including gastric cancer [47]. Further, fiber fermentation by the microbiota produces short-chain fatty acids (SCFAs), primarily acetate, propionate, and butyrate. Research indicates that these SCFAs play a critical role in maintaining immune homeostasis, especially in the context of gastrointestinal tumors [48–50], which can promote differentiation, growth arrest, and apoptosis in the gastrointestinal tract [51]. In an in vitro study, butyrate and propionate, two SCFAs, induced apoptosis and necrosis in gastric cancer cells [52]. In addition, whole grains have a lower digestion rate compared to refined grains, resulting in a lower glycemic overload and related reduced postprandial concentration of plasma insulin and insulin-like growth factor 1 (IGF-1), a possible promoter in human carcinogenesis [53]. In contrast, a diet rich in refined grains can unfavorably impact circulating insulin and related IGF-1 levels. High glycemic index (GI) and glycemic load (GL) have been associated with an increased gastric cancer risk in some studies [54, 55], though evidence remains inconsistent [56]. The mechanism involving GI/GL may also explain the stronger association observed when including among refined grains variable also grain-based sweets/desserts, which typically exhibit a higher GI and GL due to their high sugar content. Additionally, grain-based sweets and desserts are high in fats, and a diet high in saturated fat was found to increase the risk of gastric cancer [57].

However, refined and whole grain consumption may reflect, respectively, general unfavorable and favorable lifestyle and dietary habits. Indeed, a diet characterized by a high intake of refined grains may be low not only in whole grains and dietary fiber, but also in fruits, vegetables, and related beneficial micronutrients and bioactive compounds. Conversely, diets rich in whole grains are generally associated with healthier dietary profiles. However, allowance for tobacco, alcohol as well as intake of selected dietary factors related to gastric cancer risk (such as fruit, vegetable and salt), did not account for the associations observed. Adjustment for total energy intake attenuated, but did not fully explain, the association between refined grain consumption and gastric cancer risk, suggesting a role of refined grains beyond their contribution to overall energy intake.

Among the limitations, studies included may suffer from selection and recall bias. Additionally, the FFQs were not specifically designed to assess grain intake but to be representative of each country’s diet. Regarding whole grains, in particular, the FFQs included from 1 to 4 whole grain foods items. Accurate and precise quantification of whole grain intake is further hampered by the fact that, while some foods are composed entirely of whole grains (e.g., brown rice), others contain whole grains and non–whole grain ingredients, including refined grains, in varying proportions (e.g., bread made with a mixture of whole and refined flour). There is large variation across countries and organization in the definition of whole grain foods when the product is not 100% whole grain, based on the proportion of whole grain content or on a minimum amount of whole grains per serving [58]. However, there was no clear definition of whole grain foods in the FFQs to allow more precise quantification of the individual intake of whole grains. In any case, misclassification of whole and refined grain intake due to both recall bias and the limitations of FFQs is likely to be non-differential with respect to case–control status, probably leading to underestimate of the associations. As an additional limitation, adjustment for *H. pylory* infection was possible only in a subset of studies. However, the similarity of results after such adjustment suggests that *H. pylori* was not a major confounder of the association. Further, we adopted a two-stage approach to analyze data, and found a moderate to high degree of heterogeneity across studies. Notwithstanding, the present work has various advantages. First, the analysis was based on data from several studies conducted in different geographic areas and which were harmonized centrally at the StoP Project coordinating center using a common methodology. A further strength is that, in most of the studies, we were able to adjust our estimates for several potential confounders, such as smoking, alcohol drinking, fruit, vegetable and salt intake, and total energy intake; residual confounding by lifestyle and dietary habits is however possible.

In conclusion, the present analysis, based on a unique pool of data from studies conducted in populations with different patterns of grain consumption, found a differential role of whole and refined grains in gastric cancer risk, with whole grains being inversely associated and refined grains directly associated with risk. Thus, our findings support dietary recommendations favoring the intake of whole grains over refined grains [59–61].

## Supplementary Information

Below is the link to the electronic supplementary material.

Supplementary materials

## Data Availability

Data described in the manuscript, code book, and analytic code will be made available upon request from the corresponding author pending approval of the Steering committee and the participating study centers of the StoP Project.
