# Supplementary material for: Consumption of whole and refined grains and the risk of gastric cancer: a pooled analysis within the Stomach cancer Pooling (StoP) Project
Source: Eur J Nutr. 2026 Jun 13;65(4):162. doi: 10.1007/s00394-026-04003-w (PMC13264596; doi:10.1007/s00394-026-04003-w)

Supplementary Materials

Consumption of Whole and Refined Grains and the Risk of Gastric Cancer: A Pooled Analysis within the Stomach Cancer Pooling (StoP) Project

European Journal of Nutrition

Turati F, Bravi F, Pelucchi C, Bonzi R, Johnson KC, Hu J, Ferraroni M, Zhang ZF, Yu G, Lunet N, Morais S, Zaridze D, Maximovitch D, Vioque J, Gonzalez-Palacios S, Curado MP, Dias-Neto E, Castaño-Vinyals G, Fernández de Larrea-Baz N, López-Carrillo L, Hernández-Ramirez RU, Tsugane S, Hamada GS, Ward MH, Mu L, Rabkin CS, Hidaka A, Lagiou A, Lagiou P, López-Cervantes M, Boffetta P, Camargo MC, Boccia S, Negri E, La Vecchia C.

Corresponding author: Bravi Francesca, Department of Clinical Sciences and Community Health, University of Milan; email: francesca.bravi@unimi.it

Supplementary Table 1. Characteristics of the studies included in the current analysis, including whole grain food items in the food frequency questionnaires (FFQ) and availability of data on total energy intake (TEI) and *Helicobacter pylory* (*H. pylori*) infection.

StoP study ID	Study (Reference)	Period	Cases n (%)	Controls n (%)	Study type	Whole grain FFQ items	Availability of TEI/ *H. pylori*
	Europe						
1	Italy 1 (La Vecchia et al., 1995)	1985-1997	769 (9.7)	2081 (10.6)	CC, HB	1 item: Any whole grain product	No / No
3	Italy 2 (Lucenteforte et al., 2008)	1997-2007	230 (2.9)	547 (2.8)	CC, HB	1 item: Whole grain bread	Yes / No
5	Italy 4 (Buiatti et al. 1989)	1985-1987	1016 (12.8)	1159 (5.9)	CC, PB	1 item: Whole grain bread	Yes / No
6	Greece (Lagiou et al., 2004)	1981-1984	110 (1.4)	100 (0.5)	CC, HB	1 item: Rye bread	Yes / No
17	Portugal (Lunet et al. 2007)	1999-2006	692 (8.7)	1667 (8.5)	CC, PB	1 item: Brown or whole wheat bread or toasts	Yes / Yes
9	Russia (Zaridze et al., 2000)	1996-1997	450 (5.7)	611 (3.1)	CC, HB	3 items: Oat porridge; millet gruel; buckwheat gruel	Yes / Yes
21	Spain 1^ (Castaño-Vinyals, 2015)	2008-2012	339 (4.3)	3040 (15.4)	CC, PB	4 items: Whole wheat bread; wholegrain cereals; other wholegrain products; wholegrain biscuits	Yes / Yes
23	Spain 2 (Santibanez et al., 2012)	1995-1999	401 (5.1)	455 (2.3)	CC, HB	1 tem: Whole grain bread	Yes / No[§]
	Asia						
2	China 1 (Deandrea et al., 2010)	1987-1989	266 (3.4)	533 (2.7)	CC, HB	NA	No / No
8	China 2 (Mu et al., 2005)	2000	206 (2.6)	415 (2.1)	CC, PB	1 item: Barley porridge	No / Yes
12	China 3 (Setiawan et al., 2005)	1991-1993	711 (9.0)	711 (3.6)	CC, PB	NA	Yes / No
30	Japan (Machida-Montani et al., 2004)	1998-2002	153 (1.9)	303 (1.5)	CC, HB	NA	Yes / Yes
	North America						
7	Canada (Mao et al., 2002)	1994-1997	1182 (14.9)	5039 (25.5)	CC, PB	3 items: Dark bread; granola; cooked cereals	No / No
32	USA 2 (Ward et al., 1997)	1988-1993	170 (2.1)	502 (2.5)	CC, PB	2 items: Dark bread; cooked cereals	Yes / No
	Latin America						
25	Mexico 1 (Hernandez-Ramirez et al., 2009)	2004-2005	248 (3.1)	478 (2.4)	CC, PB	NA	No / Yes
26	Mexico 2 (Lopez-Carrillo et al., 1994)	1989-1990	220 (2.8)	752 (3.8)	CC, PB	NA	No / No
27	Mexico 3 (Lopez-Carrillo et al., 2003)*	1994-1996	93 (1.2)	186 (0.9)	CC, HB	3 items: Whole grain bread; whole grain cereals; oat	Yes / Yes
28	Brazil 1 (Nishimoto et al., 2002)	1991-1994	226 (2.9)	226 (1.2)	CC, HB	NA	No / Yes
29	Brazil 2 (Hamada et al., 2002)	1991-1994	93 (1.2)	186 (0.9)	CC, HB	NA	No / Yes
36	Brazil 3 (Bartelli et al., 2019)	2016-2020	368 (4.6)	738 (3.7)	CC, HB	4 items: Whole grain bread; oat; brown rice; pop corn	No / Yes

CC, case-control; HB, hospital-based; PB, population-based. NA, not available; TEI, total energy intake. * Of the 234 cases and 468 controls recruited in the study, we included 93 cases and 186 controls with available dietary data. ^ Of the 441 cases and 3441 controls recruited in the study, we included 339 cases and 3040 controls with available dietary data. [§]Available only for a small fraction of subjects.

Supplementary Figure S1. Participant flow chart.

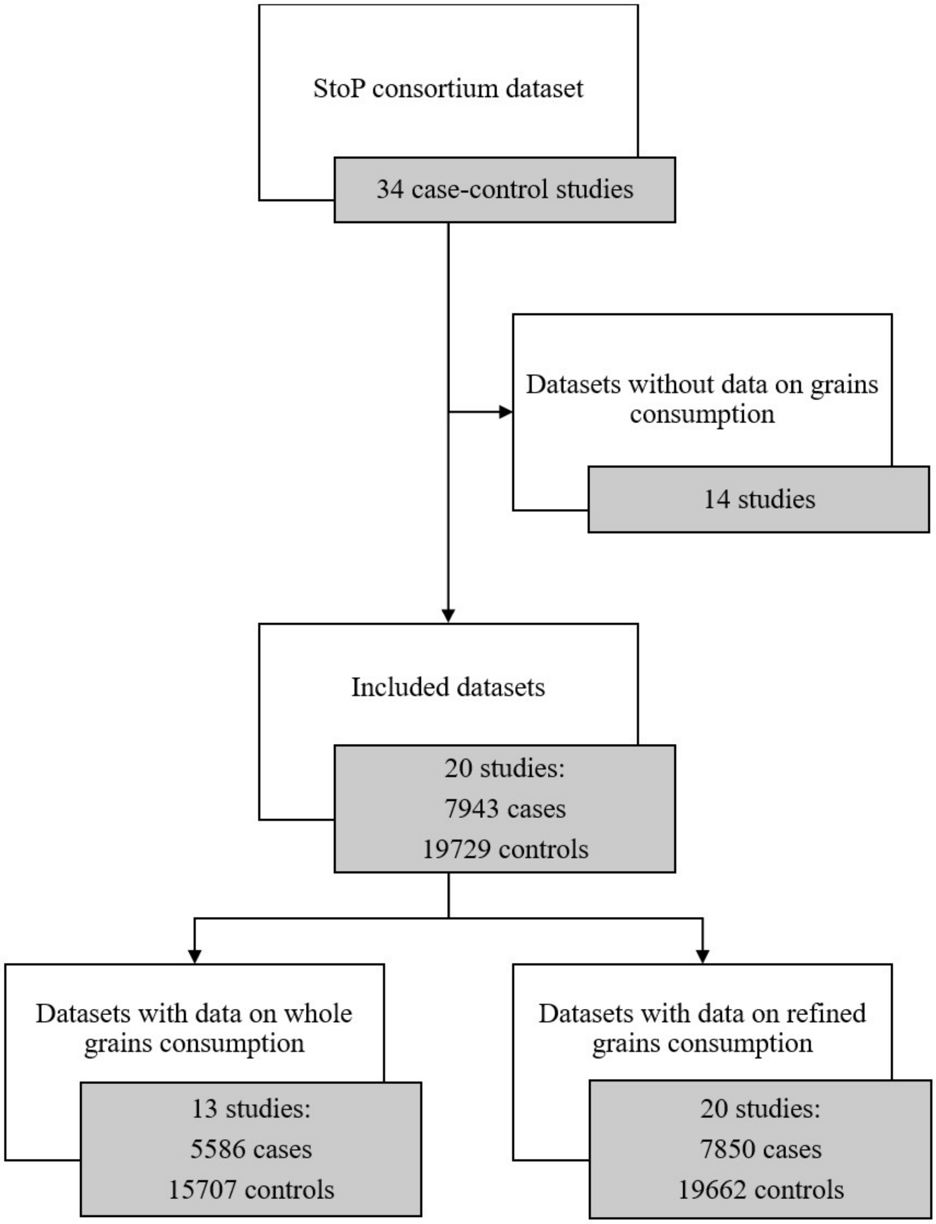

Supplementary Figure S2. Influence analysis showing pooled estimates after removing one study at a time from the two-stage procedure for the highest versus the lowest category of consumption of whole grains (i.e., ≥ median consumption versus no consumption, A), refined grains (third versus first tertile, B), and refined grains including sweets (third versus first tertile, C).

A - Whole grain intake: ≥ median vs none

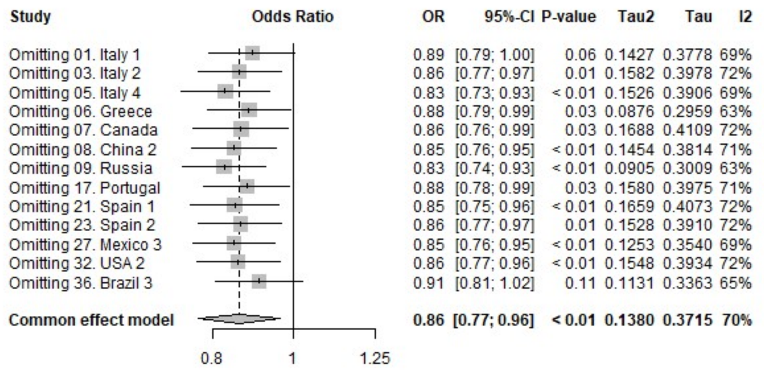

B - Refined grain intake: T3 vs T1

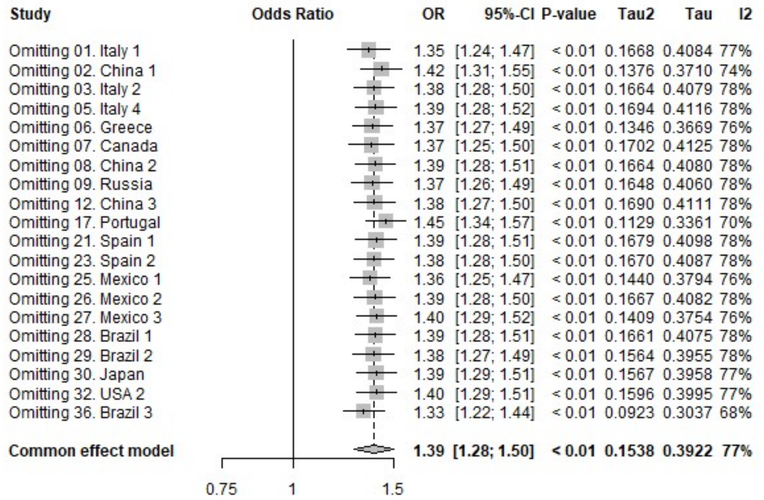

C - Refined grain including sweets intake: T3 vs T1

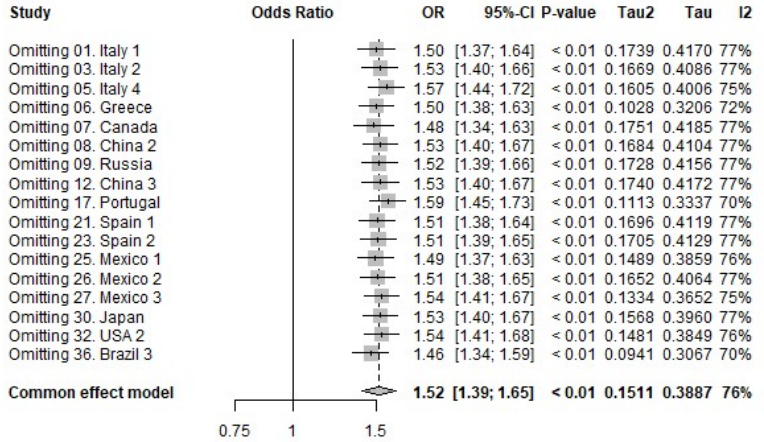

Supplement: Supplementary file 1 — Supplementary materials [file 394_2026_4003_MOESM1_ESM.pdf]
